# Rapid Response to Ebola Outbreaks in Remote Areas — Liberia, July–November 2014

**Published:** 2015-02-27

**Authors:** Francis Kateh, Thomas Nagbe, Abraham Kieta, Albert Barskey, Alex Ntale Gasasira, Anne Driscoll, Anthony Tucker, Athalia Christie, Ben Karmo, Colleen Scott, Collin Bowah, Danielle Barradas, David Blackley, Emmanuel Dweh, Felicia Warren, Frank Mahoney, Gabriel Kassay, Geoffrey M. Calvert, Georgina Castro, Gorbee Logan, Grace Appiah, Hannah Kirking, Hawa Koon, Heather Papowitz, Henry Walke, Isaac B. Cole, Joel Montgomery, John Neatherlin, Jordan W. Tappero, Jose E. Hagan, Joseph Forrester, Joseph Woodring, Joshua Mott, Kathleen Attfield, Kevin DeCock, Kim A. Lindblade, Krista Powell, Kristin Yeoman, Laura Adams, Laura N. Broyles, Laurence Slutsker, Lawrence Larway, Lisa Belcher, Lorraine Cooper, Marjorie Santos, Matthew Westercamp, Meghan Pearce Weinberg, Mehran Massoudi, Monica Dea, Monita Patel, Morgan Hennessey, Moses Fomba, Mutaawe Lubogo, Nikki Maxwell, Patrick Moonan, Sampson Arzoaquoi, Samuel Gee, Samuel Zayzay, Satish Pillai, Seymour Williams, Shauna Mettee Zarecki, Sheldon Yett, Stephen James, Steven Grube, Sundeep Gupta, Thelma Nelson, Theophil Malibiche, Wilmont Frank, Wilmot Smith, Tolbert Nyenswah

**Affiliations:** 1 Liberia Ministry of Health and Social Welfare, Monrovia, Liberia; 2 CDC; 3 World Health Organization, Geneva, Switzerland; 4 United Nations Children’s Fund, New York City, New York; 5 African Union, Addis Ababa, Ethiopia

West Africa is experiencing its first epidemic of Ebola virus disease (Ebola) (1). As of February 9, Liberia has reported 8,864 Ebola cases, of which 3,147 were laboratory-confirmed. Beginning in August 2014, the Liberia Ministry of Health and Social Welfare (MOHSW), supported by CDC, the World Health Organization (WHO), and others, began systematically investigating and responding to Ebola outbreaks in remote areas. Because many of these areas lacked mobile telephone service, easy road access, and basic infrastructure, flexible and targeted interventions often were required. Development of a national strategy for the Rapid Isolation and Treatment of Ebola (RITE) began in early October. The strategy focuses on enhancing capacity of county health teams (CHT) to investigate outbreaks in remote areas and lead tailored responses through effective and efficient coordination of technical and operational assistance from the MOHSW central level and international partners. To measure improvements in response indicators and outcomes over time, data from investigations of 12 of 15 outbreaks in remote areas with illness onset dates of index cases during July 16–November 20, 2014, were analyzed. The times to initial outbreak alerts and durations of the outbreaks declined over that period while the proportions of patients who were isolated and treated increased. At the same time, the case-fatality rate in each outbreak declined. Implementation of strategies, such as RITE, to rapidly respond to rural outbreaks of Ebola through coordinated and tailored responses can successfully reduce transmission and improve outcomes.

Outbreaks in remote areas posed a significant challenge to CHTs to mount an effective investigation and rapid response because of limited resources, personnel, and means to reach remote areas. The RITE strategy provided a framework to coordinate assistance from the central MOHSW and other agencies under the leadership of the CHT and developed several tools to help plan, manage, and track a response effort. The objectives of the investigation and response teams were to 1) rapidly isolate and treat Ebola patients, either by establishing isolation and treatment facilities in the community or by safely transporting patients to existing Ebola treatment units (ETUs); 2) ensure proper collection and safe transportation of samples for Ebola laboratory confirmation; 3) ascertain the index case (the first person in the transmission chain who entered the community from another county in Liberia) in each outbreak to better understand importation and transmission patterns; 4) identify all generations of cases by improving case finding and contact tracing to ensure no cases were missed; 5) train teams in safe burial procedures; and 6) observe contacts for 21 days from the death or ETU admission of the last case to ensure interruption of transmission. Investigation and response teams included Liberian MOHSW national and county representatives, CDC, WHO, the United Nations Children’s Fund, and other multilateral and nongovernmental organizations.

The RITE strategy clearly articulated the role of CHTs to coordinate efforts of partners involved in response activities to rapidly mobilize resources that could be tailored to the needs in each outbreak. After initiation of the RITE strategy in October, outbreak responses were supported with structured rapid response microplanning tools implemented by CHTs that delineated each intervention component (e.g., isolation of patients, laboratory testing, and health promotion) and the organizations responsible for implementation. Outbreaks and response activities were reviewed on a weekly basis at the national level at the county operations subcommittee of the national incident management system (2), allowing MOHSW and partner organizations to plan and prioritize resources for the rapidly changing situation. An additional intervention beginning in November was the packaging of RITE kits that could be prepositioned in the counties, containing all commodities required for the first 14 days of response interventions (e.g., essential medicines for treatment of patients such as oral rehydration solution, antimalarial medication, and antibiotics; personal protective equipment; and construction materials for temporary isolation and treatment facilities). The availability of RITE kits at the county level would provide further flexibility to CHTs to tailor rapid responses appropriately for the community involved in the outbreak and add to their ability to rapidly deploy the necessary resources to the affected area.

For this report, Ebola outbreaks that occurred in remote areas, produced at least one generation of transmission in the community and had complete investigations were analyzed. An Ebola outbreak was defined as two or more epidemiologically linked Ebola cases. Cases were classified as suspected, probable, or confirmed using the Liberian case definitions (3).

Initial alerts of possible Ebola outbreaks were received by CHTs as rumors, reports of clusters of ill persons or unexplained deaths, or reports of patients admitted to ETUs. Case investigation reports were gathered through interviews with ill persons or their proxies. Databases from ETUs were searched to supplement incomplete case investigation reports. Transmission-chain diagrams were constructed back to the first case to enter the county from another county in Liberia (the index case). The first generation of cases was defined as resulting from contact with the index patient, and the total number of generations was determined from the transmission-chain diagrams. To monitor the effectiveness of rapid response to outbreaks over time, the number of days between the symptom onset of the index patient and the date the CHT was first alerted to a potential outbreak, and the date the CHT first sent in a team to investigate were computed for each outbreak. Outbreak duration was calculated as the number of days between the symptom onset date of the index case and the last case in the outbreak, defined as the last case in a chain of transmission to occur before 21 days passed with no new cases. Demographic characteristics of patients and the proportion of patients isolated and treated in an ETU or similar facility were summarized by outbreak.

Among 15 Ebola outbreaks in remote areas of nine counties with index case symptom onset dates during July 16–November 20, 2014, 12 investigations had complete data (Figure 1). Investigations of these 12 outbreaks identified 263 patients (Table), including 155 (59%) with laboratory-confirmed cases of Ebola, 71 (27%) with probable cases, and 37 (14%) with suspected cases. There were 190 deaths (case-fatality rate = 72%). The median number of cases in an outbreak was 22 (range = 4–64), and the median population in the affected communities was 800 (range = 301–6,200). Attack rates ranged from 1 to 71 cases per 1,000 population. The median age of the patients was 32 years (range = 15 days to 84 years), and 144 (55%) were female. Eight (67%) outbreaks began with the introduction of an Ebola case from Monrovia, two from a neighboring county, and the source of introduction for three outbreaks was not identified (one outbreak had two index cases) (Table). Transmission of Ebola occurred through close contact with persons who were ill with Ebola, including providing care to a patient at home, or contact with a person who had recently died from Ebola. In Small Ganta, Nimba County, the death and burial of a woman who cared for the index patient resulted in 16 (25%) of the 64 Ebola cases in that outbreak. Although several traditional healers were infected in these outbreaks, no cases in health care workers from public or private health facilities were identified.

The median time between symptom onset in the index patient and an alert received by the CHT was 33 days (range = 0–58 days); the median time to alert was 40 days for the six outbreaks before October 1 (prior to initiation of the RITE strategy) and 25 days for the six outbreaks with onset after October 1 (after the RITE strategy) (Figure 2). The median duration of the outbreaks was 43 days (range = 7–97 days). The median duration of the early outbreaks was 53 days, compared with 25 days for the later outbreaks. The median number of generations of cases was four (range = 1–7) for the early outbreaks and two (range = 1–4) for the later outbreaks.

Interventions in the 12 outbreaks included 1) engagement of traditional and community leaders in response activities; 2) community education about Ebola virus transmission and prevention; 3) active case finding, contact tracing and monitoring; 4) quarantine of asymptomatic high risk contacts at home or in designated quarantine facilities; 5) isolation and treatment of patients; and 6) safe burials. In each community, the appropriate level of intervention was determined by the community’s requests, the number and severity of cases, the remoteness and accessibility of the community, and the distance to Ebola treatment facilities. Resistance to assistance was encountered in several communities, and response was suspended until discussions with county and traditional officials or the increasing burden of cases and deaths encouraged community acceptance of intervention. In two outbreaks (Kayah District, Rivercess and Quewein, Grand Bassa), the availability of nongovernmental partners to rapidly establish isolation and treatment facilities permitted on-site or nearby care of patients. In these and other outbreaks, some patients were able to reach ambulances at the closest road access point and were taken to established ETUs. In one outbreak (Jenewonde, Grand Cape Mount), delays in the establishment of an isolation and treatment facility resulted in only one patient being cared for in the facility before the outbreak was over.

Over time, the proportion of patients in each outbreak that were isolated and treated increased from a median of 28% in the early outbreaks to 81% in the later outbreaks (Table). The proportion of laboratory-confirmed cases increased from a median of 44% in the early outbreaks to 81% in the later outbreaks because a greater proportion of patients reached treatment facilities and specimen collection in the field improved as part of the RITE strategy. The case-fatality rate of each outbreak also improved over time; the median case-fatality rate in the early and later outbreaks were 87% and 50%, respectively. As of January 8, 2015, all of these outbreaks had ended with no further cases identified within 21 days of exposure to the last patient.

## Discussion

**FIGURE 3:**
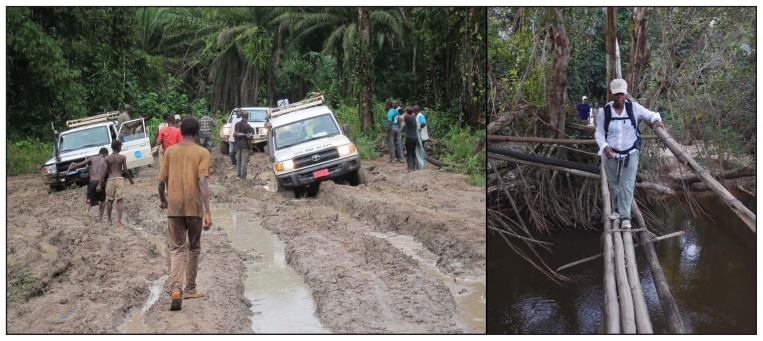
Challenges facing Ebola teams traveling to remote communities in Liberia have included impassable roads such as this one on the way to John Logan Town (left) and difficult river crossings such as this one on the way to Bomota (right) Photo/Justin Williams Photo/Sampson Dolo

Implementing an effective rapid response is critical to limiting the magnitude and duration of Ebola outbreaks. The remoteness and complexity of the outbreaks described in this report have posed challenges to rapid response; movement of personnel and supplies was greatly hindered by distance, river crossings, poor or nonexistent roads (Figure 3), and limited communication options (4). Nonetheless, implementation of the RITE strategy resulted in substantial reductions in the time from symptom onset of the index patient to outbreak alerts, in the duration of outbreaks, and in the case-fatality rate.

Four of the six outbreaks that occurred before development of the RITE strategy remained undetected until they were in at least the third generation of transmission, whereas five of the six later outbreaks were detected in the first or second generation. In addition to the RITE strategy, greater community awareness of Ebola helped alert authorities earlier to clusters of unexplained deaths or illness consistent with Ebola and also facilitated faster community acceptance of interventions. Availability of ETU beds for isolation and treatment of patients also improved significantly over the period covered by these outbreaks (5), and the increasing proportion of patients reaching an ETU or other type of isolation and treatment facility likely contributed to the shorter duration of outbreaks. Continued access to early treatment, efforts to improve community awareness, and deployment of rapid, coordinated, and effective responses to remote rural outbreaks will need to continue until Ebola transmission in West Africa is halted.

What is already known on this topic?

The epidemic in West Africa has resulted in the largest number of Ebola cases in history. Ebola is associated with a high case-fatality rate that can be reduced through supportive care. Ebola transmission can be interrupted through isolation of infected patients, infection control, monitoring of patients’ contacts, and safe burial of dead bodies. Remote rural areas pose challenges for rapid isolation and treatment of patients because of their distance, difficult access, and lack of communications infrastructure.

What is added by this report?

A national strategy in Liberia to coordinate rapid responses to remote outbreaks of Ebola reduced by nearly half the time between the first new case in remote areas and notification of health authorities. As coordination of the rapid response strategy improved over time, the median duration of outbreaks decreased from 53 to 25 days as the number of generations of cases decreased from a median of four to two. The proportion of patients isolated increased from 28% to 81%; survival improved from 13% to 50%.

What are the implications for public health practice?

Ebola outbreaks in remote rural areas require rapid responses, including the movement of patients to treatment facilities. Interventions can be as simple as arranging safe ambulance transport for patients who might have to walk out of remote areas, but might also require establishment of mobile isolation and treatment facilities if patients are too ill to move or delays in transport are anticipated. Comprehensive and innovative rapid response units can improve outcomes and shorten duration of Ebola outbreaks, and should be employed wherever possible.

## Figures and Tables

**FIGURE 1:**
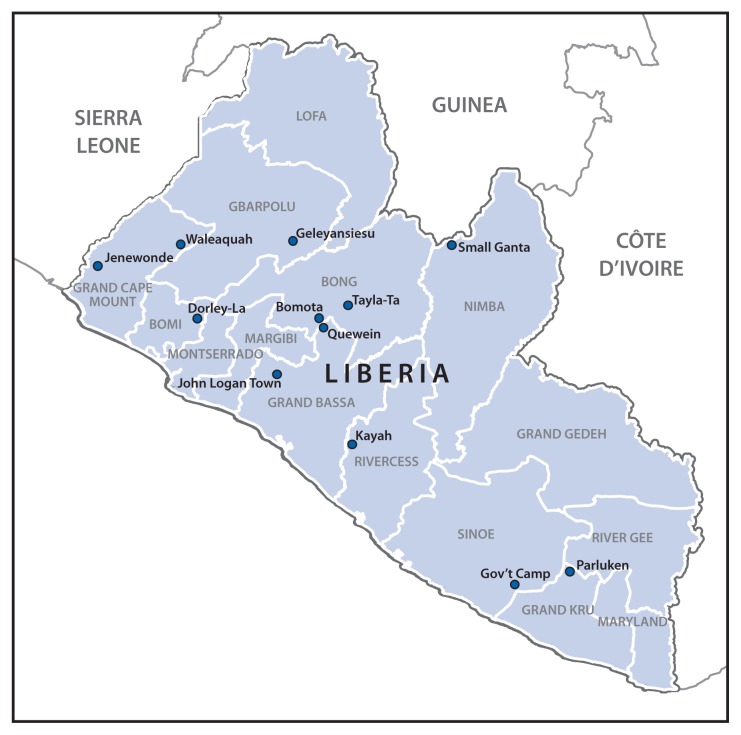
Locations of 12 Ebola outbreaks in remote communities — Liberia, July 16–November 20, 2014

**FIGURE 2:**
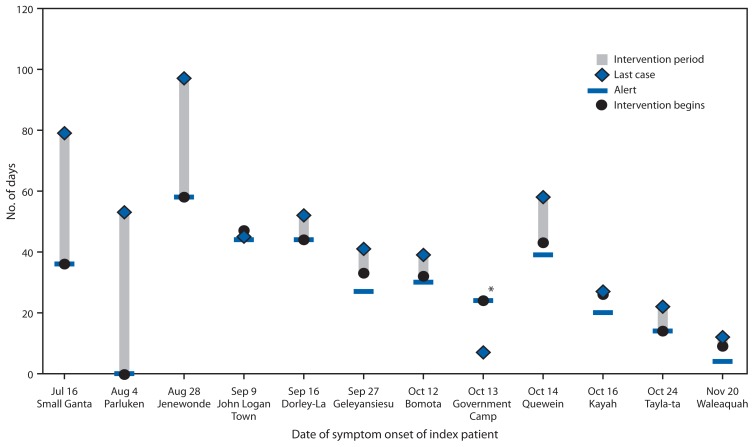
Number of days from Ebola symptom onset of the index patient to alert of the county health team, beginning of intervention, and the last reported case in 12 Ebola outbreaks in remote communities — Liberia, July 16–November 20, 2014 * The alert to the county health team did not come until after the last reported case on October 20. The investigation, which included contact monitoring, began on November 6.

**TABLE:** Characteristics of 12 Ebola outbreaks in remote communities — nine counties, Liberia, July 16–November 20, 2014

County	Community	Estimated pop.	Date of symptom onset of index case	Origin of index case	Days from index case onset to investigation	Total no. of cases (% laboratory-confirmed*)	Estimated attack rate (per 1,000 pop.)	% female	% isolated and treated	Case-fatality rate (%)
Nimba	Small Ganta	900	July 16	Monrovia	36	64 (47)	71	64	25	73
Grand Kru	Parluken	2,000	August 4	River Gee	0	17 (47)	9	41	18	82
Grand Cape Mount	Jenewonde	800	August 28	Monrovia	58	35 (40)	44	51	31	91
Grand Bassa	John Logan Town	5,000	September 9	Unknown	47	17 (12)	3	29	6	94
Bomi	Dorley-La	301	September 16	Monrovia	44	10 (40)	33	50	50	100
Gbarpolu	Geleyansiesu	800	September 27	Kakata, Margibi	33	22 (82)	28	41	68	73
Bong	Bomota	397	October 12	Unknown	32	14 (86)	35	57	86	50
Sinoe	Government Camp	6,200	October 13	Monrovia	24	4 (75)	1	25	75	50
Grand Bassa	Quewein	371	October 14	Monrovia	43	24 (75)	65	65	54	61†
Rivercess	Kayah	5,000	October 16	Monrovia	26	22 (59)	4	64	41	59
Bong	Tayla-ta	500	October 24	Monrovia	14	28 (96)	56	68	96	50
Grand Cape Mount	Waleaquah	700	November 20	Monrovia	9	6 (100)	9	33	100	50

* Laboratory confirmation of cases was performed by real-time polymerase chain reaction testing at one of four laboratories in Liberia.

† One patient died from an accident and was not included in the case-fatality rate calculation.
